# Risk assessment and predation potential of *Stratiolaelaps scimitus* (Acari: Laelapidae) to control *Varroa destructor* (Acari: Varroidae) in honey bees

**DOI:** 10.1371/journal.pone.0208812

**Published:** 2018-12-07

**Authors:** Sabrina Rondeau, Pierre Giovenazzo, Valérie Fournier

**Affiliations:** 1 Département de phytologie, Université Laval, Québec City, Quebec, Canada; 2 Département de biologie, Université Laval, Québec City, Quebec, Canada; University of North Carolina at Greensboro, UNITED STATES

## Abstract

The biocontrol of the honey bee ectoparasite *Varroa destructor* is an underexploited but promising avenue that would benefit from being integrated in a *Varroa* management program. Our study aimed to investigate the potential of the predatory mite *Stratiolaelaps scimitus* to control *Varroa* infestations in honey bees. Tests on safety and predation were carried out to: (1) assess the risk of predation of the honey bee brood by *S*. *scimitus* under laboratory conditions and within the colony, and (2) evaluate the predation potential of *S*. *scimitus* on phoretic *Varroa* mites. Under laboratory conditions, *S*. *scimitus* was able to feed upon free *Varroa* mites, but also attacked every unprotected honey bee brood stages with a strong preference for bee eggs. When introduced inside colonies, however, *S*. *scimitus* does not have negative effects on the survival of the bee brood. Moreover, observations made in the laboratory revealed that *S*. *scimitus* does not attack *Varroa* mites when they are attached to the body of bees. However, all *Varroa* mites that had naturally fallen from the bees were predated upon by *S*. *scimitus* and died in less than 24h. This study provides evidence that *S*. *scimitus* does not represent a significant threat to the bee brood, but also suggests that its effect in *Varroa* control will probably be limited as it does not attack phoretic *Varroa* mites. Our results represent a first step in assessing the potential of *S*. *scimitus* to control *V*. *destructor* and provide novel information about the predator’s behavior inside the honey bee colony.

## Introduction

The ectoparasitic mite *Varroa destructor* Anderson & Trueman (Acari: Varroidae) is considered as the most damaging honey bee (*Apis mellifera* L.) pest worldwide [1, 2]. Since its introduction in Europe in the 1970s and in North America in the 1980s [3], the *Varroa* mite has caused major damages and economic losses to the beekeeping industry [4, 5]. In North temperate regions of America and much of Europe, the pest is also a key factor of high winter colony losses [6–8]. Through direct physical damages to honey bees [3, 9] and transmission/activation of many honey bee viruses [10–12], an untreated infested colony will most likely die within months [13].

Controlling *Varroa* mite populations in honey bee colonies is challenging as there exists no one-fits-all approach to get rid of the pest. Even though synthetic acaricides have been successfully used for *Varroa* control in the past years [14], the development of mite resistance now limits their use [15–17]. As alternative treatments, some “natural chemicals” such as organic acids and essential oils are increasingly used by beekeepers but also have disadvantages such as variable toxic effect on bees [18–22], possible contamination of wax and honey [23, 24] and an effectiveness dependent on environmental conditions [25]. Thus, Integrated Pest Management (IPM), which combines non-chemical and chemical methods with *Varroa* infestation thresholds, is currently considered as the best approach to control the *Varroa* and aims to reduce beekeepers’ reliance on synthetic acaricides [3, 26, 27].

The biocontrol of *Varroa* mites is an underexploited but promising avenue that could enhance an IPM strategy. Despite all the known benefits of the biological pest control, little research has been done on the use of living organisms to control *Varroa* mites. In addition to be lethal for *Varroa* mites, a good candidate biocontrol agent should have: (1) the ability to operate under the physical conditions of a honey bee colony, (2) the ease of targeting against the *Varroa*, and (3) the potential for mass production [28]. According to Chandler et al. [28], as *V*. *destructor* seems to be relatively free of natural enemies, its biocontrol is likely to require natural enemies from other hosts. Likewise, the absence of identified specialist enemies of *Varroa* mites [29] brings us to consider generalist predators as potential biocontrol agents.

Due to its ecology and specific characteristics, the predatory mite *Stratiolaelaps scimitus* (Womersley) (Acari: Laelapidae), formerly known as *Hypoaspis miles* (Berlese), appears to be particularly promising as a biocontrol agent against *Varroa* mites. *Stratiolaelaps scimitus* is a polyphagous soil-dwelling mite naturally occurring throughout the Northern hemisphere [30]. It preys upon many soil organisms such as thrips nymphs, nematodes, phorid and sciarid fly larvae and several species of mites and other invertebrates [31–33]. The predatory mite thrives in hot and humid environments and can survive temperatures up to 32°C [34], which suggests its adaptability to the conditions observed within a honey bee colony. Already mass-reared and commercially available in North America and Europe [32], *S*. *scimitus* has proven to be useful in the biocontrol of fungus gnats and thrips of protected crops [35–39] and is now known to reduce infestations of the poultry red mite on chicken livestock in small cages [40]. More recently, the pet industry has also started using *S*. *scimitus* as a means to control parasitic mites on reptiles in captivity [41] although little data is available on the actual effectiveness of this practice.

Nowadays, some beekeepers in the United States, Canada and Europe are using *S*. *scimitus* for *Varroa* mite control in honey bee colonies but to date, no scientific study has shown the effectiveness of the investigated biocontrol agent to control *Varroa* populations *in situ*. A team of researchers from Texas (USA) has recently demonstrated, using *in vitro* trials, that *S*. *scimitus* indeed attacks and feeds upon free *Varroa* mites [42]. However, little is known about its effectiveness in the hive and while some anecdotal observations made in Ontario (Canada) suggest that *S*. *scimitus* would reduce *Varroa* mite populations when introduced in honey bee colonies [43], a similar field experiment resulted in ineffective *Varroa* control [42]. Despite these contradictory results and the lack of experimental proof of effectiveness, some biocontrol suppliers are now selling *S*. *scimitus* for *Varroa* control. Considering that effective *Varroa* control is a key factor for honey bee colony survival [44], the use of a method whose real effectiveness is unknown could have detrimental consequences for the apiarists’ bee stocks and the beekeeper’s perception of biocontrol.

Before demonstrating the impact of *S*. *scimitus* in *Varroa* biocontrol inside the honey bee colony, it is judicious to test its safety and predation effectiveness in lab bioassays. Indeed, as previously put forward by Chandler et al. [28], there is a significant risk that any generalist predator introduced in a colony as a means of *Varroa* control would consume bee eggs. Another important factor to consider is that to be effective, the predator must be able to attack phoretic *Varroa* mites and not just the free mites. Free *Varroa* mites are not common in a bee colony as the mites are found either attached to the body of an adult bee (phoretic stage) or parasitizing a pupa in a capped brood cell (reproductive stage) [5, 45]. Therefore, as *S*. *scimitus* cannot reach reproducing *Varroa* mites because they are protected by a wax cap, it must attack those parasitizing adult bees for the treatment to be effective.

Our study aimed to investigate the potential of *S*. *scimitus* to control *Varroa* mite infestations in honey bees. The specific objectives of this paper were: (1) to assess the risk of predation of honey bee brood by *S*. *scimitus* under both laboratory conditions and within the colony, and (2) to evaluate the predation potential of *S*. *scimitus* on phoretic *Varroa* mites. According to what we know from the literature, we hypothesized that the use of *S*. *scimitus* in *Varroa* biocontrol would not be a threat to the honey bee brood. In fact, the bee brood does not correspond to the type of prey typically consumed by *S*. *scimitus* [34, 39]. We also believe that *S*. *scimitus* is a potential predator of phoretic *Varroa* mites. This hypothesis is supported by the use of the predatory mite to control hematophagous mites in infested animals [40, 46] and the few anecdotal reports by beekeepers of *Varroa* population reductions. Assessing both the risk and the predation potential of *S*. *scimitus* to control *Varroa* mites is a very important step in the study of this biocontrol agent in beekeeping.

## Materials and methods

### Livestock sources and maintenance

*Stratiolaelaps scimitus* was obtained from Applied Bio-nomics Ltd. (British Columbia, Canada). Mites were supplied in a mixture of vermiculite and peat in 1L bottles with mold mites (*Tyrophagus putrescentiae*) as a food source. The predatory mites were stored in their original containers, lying on their side in complete darkness at 15°C, and were regularly checked for predator vitality (i.e., normal activity, vigour and abundance when observed under a stereomicroscope) and the presence of prey.

Adult female *Varroa* mites were collected from infested hives located in apiaries of various beekeepers near Quebec City (Quebec, Canada) following the “Icing Sugar” method described in Dieteman et al. [1]. Briefly, we collected approximately 300 bees (125 ml) from brood frames and placed them in a 500 ml Mason jar whose lid had been replaced by a 2 mm hardware mesh. Powdered sugar (15 ml) was added through the mesh and the jar was rolled to cover the bees with sugar. After letting the jar stand for one minute, it was turned upside down and shake over a white plastic cardboard until the mites stopped falling. The mites were collected with a fine paint brush and brought to the lab. They were then maintained alive by confining them by groups of five on a drone pupa in a 1 ml Eppendorf tube pierced with two holes for ventilation and kept in an incubator (32.0 ± 0.5°C, ≈70% RH, complete darkness). *Varroa* mites were successfully kept this way for up to one week.

Honey bee (*Apis mellifera*) brood was sampled from a single hive located in the city of Levis (46°44'56.02"N, 71°10'2.17"O), 15 km from our laboratory at the Université Laval. Eggs and larvae were gently sampled with a small paintbrush and transferred in a small Petri dish (50 x 12 mm) containing a moistened filter paper. Capped pupae cells were carefully cut with a scalpel directly from brood frames and transferred to the same Petri dish. Only worker brood was used. Samples were quickly transferred into an incubator and maintained under controlled conditions (32.0 ± 0.5°C, ≈70% RH, complete darkness) until their transfer in the arenas.

Adult worker bees were collected from the livestock of a bee research facility in Quebec (Centre de recherche en sciences animales de Deschambault, CRSAD, 46°43'6.00"N, 71°33'5.79"O) and were used immediately following their collection. Similarly, all the colonies used in our study were operated by the CRSAD.

### *In vitro* assessment of *S*. *scimitus* predation upon *V*. *destructor* and bee brood

The tests took place between July 21 and September 1, 2016. There were six treatments representing potential prey for *S*. *scimitus*: 1) adult female *Varroa* mite; 2) honey bee egg; 3) 1^st^ or 2^nd^ bee larval instar (L1-L2); 4) 3^rd^ or 4^th^ bee larval instar (L3-L4); 5) 5^th^ bee larval instar (L5); and 6) capped bee pupa. Honey bee larval instars were estimated from visual assessment of the space occupied by the larva in the brood cell according to Human et al. [47], allowing for a rough estimate of age (two-instar overlap).

Experimental arenas consisted of small glass vials (5 ml) filled with 1 cm of pre-autoclaved vermiculite and moistened with 0.3 ml of tap water. Only adult female predators were used, and each one was starved individually for 48h in small portion containers (1 oz) with a piece of moistened tissue paper prior to their transfer in the arenas. Twenty starved predators were transferred to each arena with a fine paintbrush. Then, one single prey was added according to the treatment. Vials were closed with a piece of Nitex^®^ synthetic nylon screening (105 μm) and a rubber band, allowing for ventilation while blocking mite escape. Arenas were held in an incubator (32.0 ± 0.5°C, complete darkness) throughout the duration of the tests. A saltwater pool helped to maintain the desired humidity in the incubator, which varied from 48 to 76% RH.

After 12 h, each prey was observed using a stereomicroscope and was scored as follows: alive without predation, dead without predation, alive with predation, dead with predation or fully consumed. The presence of visible wounds or missing parts (legs, antennae, cuticle parts) were considered as signs of predation. Prey viability was determined by the presence of movements when touched with a fine paintbrush. If predation did not take place after 12 h, the prey was replaced by a fresh one. Arenas were then returned to the incubator for an additional 12 h and the prey were checked one last time. At the end of the test, a count of living and dead predators was done to ensure that a reasonable number of predators was still in the arena. For each treatment, a control arena (with a prey but without predators) allowed us to observe the normal appearance of the prey in absence of predation. For each trial period (block), all six treatments and their paired control counterparts were tested simultaneously according to a randomized block design and each treatment was repeated 20 times.

### Prey preference test

In order to determine if the predatory mite will more likely attack honey bee eggs or *Varroa* mites in the first place, a prey preference test was conducted using the same experimental arenas as described above. The experiment took place in the laboratory on August 5, 12 and 19, 2016 and included 10 replicates for each date (for a total of 30 replicates). Ten starved predatory mites were transferred to each arena with one honey bee egg and one female *Varroa* mite added simultaneously. For each arena, the order of prey introduction was randomly determined. Once closed, arenas were held in an incubator (32.0 ± 0.5°C, 51–75% RH, complete darkness) throughout the duration of the test. Prey were observed under a stereomicroscope every hour for signs of predation and the test ended as soon as predation was detected. The first prey attacked was considered as a choice. In the case where both prey would have been attacked in the same one-hour observation interval, the choice would have been recorded as “equal”.

### *In vivo* assessment of *S*. *scimitus* predation upon bee brood

An in-hive predation experiment was also conducted in an apiary of the CRSAD (46°47'50.09"N, 71°43'42.50"O) on colonies of equivalent strength and having sister queens of known descent. Each colony was housed in a Langstroth commercial hive consisting of a single brood chamber (10 frames) supporting two or three honey suppers over a queen excluder. Prior to the trial, visual inspections were performed to ensure that all colonies were healthy and without signs of brood diseases. On August 9, 2017, honey bee colonies were randomly assigned to two groups with five colonies per treatment: Group 1) colonies inoculated with *S*. *scimitus*, and Group 2) untreated colonies (control). For each colony, the queen was caged on a frame with empty combs for 48h and allowed to lay eggs as described in Human et al. [47]. Then, each queen was removed from the exclusion cage and reintroduced in its colony. The position of every comb cell containing an egg was marked using a permanent marker on a transparent sheet of acetate placed on each side of the frame. Each frame was placed back to the exclusion cage to prevent further oviposition by the queen and was replaced in the middle of the brood chamber. Colonies were then inoculated by pouring 500 ml (≈ 12,500 *S*. *scimitus* individuals) of the biocontrol commercial product (Group 1) or the same amount of pre-autoclaved vermiculite (Group 2) on top of the queen excluder. For both groups, the respective substrate was poured parallel to the brood frames, so that it was partially retained by both the queen excluder and the top of the frames (S1 Fig). Some substrate inevitably fell to the bottom of the hive during inoculation, but in a negligible amount. We used 500 ml of the commercial product containing *S*. *scimitus*, which is twice the dose currently recommended by biocontrol suppliers [42, 43]. In doing so, we wanted to make sure that we used enough predators to detect a predation effect, if any, while still using a realistic amount of product as it is likely to be used in honey bee hives. Six days later, brood cells of each frame were observed for a second time by checking with previous acetates if the larvae (L4-L5) were present. Cells with a missing larva were marked with a permanent marker of another color before the combs were returned to the hives. This was repeated four days later (capped pupa). At each period, cells with brood were counted to determine the percentage of eggs and larvae that survived until cell capping. At each of the three periods of brood monitoring, hive floor and frames were also visually checked to ensure that the predatory mites remained in the hives. Observing five to ten mites during a visual inspection was considered satisfactory. At the end of the trial, a sample of debris (≈ 60 ml) was collected at the bottom of the hive for further screening under the stereomicroscope.

The number of experimental units (bee colonies) used in this trial is rather low given certain constraints related to the equipment availability and handling time. If resources are available, a better statistical power could be obtained in further studies by increasing the number of colonies under study. The full protocol is available at protocols.io (http://dx.doi.org/10.17504/protocols.io.unaevae).

### *S*. *scimitus* predation of phoretic *Varroa* mites

The experiment was conducted in the laboratory at two distinct periods, each one included half of the replicates. The first part of the trials started on July 10, 2017, while the other one started on August 9, 2017. Modified plastic pill bottles (34 mm diameter; 63 mm high) served as experimental arenas in which a hole was cut in the lid and was then covered with a glued piece of Nitex^®^ synthetic nylon screening (105 μm). A hole was cut in the lowest quarter of each bottle allowing for the insertion of a 0.5 ml Eppendorf tube pierced with three small holes and serving as a bee feeder. Paraffin film was used to ensure tightness. Bottles were filled with 5 ml of pre-autoclaved vermiculite moistened with 2 ml of tap water. In a completely randomized design, twenty starved adult female *S*. *scimitus* were transferred to each treated arena (n = 40) whereas control arenas (n = 40) received no predators.

Using a fine paintbrush, one freshly collected adult female *Varroa* mite was transferred to the body of each adult worker bee used in this trial. Then, a parasitized bee was introduced in each arena and was fed daily with a 50% (w/v) sucrose solution. Arenas were held in a growth chamber (30.0 ± 0.5°C, 75 ± 2% RH, complete darkness) throughout the duration of the test (i.e., from 1 to 14 days according to *Varroa* survival time). Once a day, honey bees and *Varroa* mites were observed and recorded as dead or alive. If the honey bee was dead but the *Varroa* was still alive, the bee was changed by a new one and the *Varroa* was transferred back on its body. For each arena, observations ended as soon as the *Varroa* was recorded dead and the latter was then observed under a stereomicroscope for evidence of predation. Here again, a count of living and dead predatory mites was done at the end of the test to ensure that a reasonable amount of living predators was still in the treated arenas.

### Data analyses

Descriptive statistics of *in vitro S*. *scimitus* predation upon *Varroa* mites and bee brood are given as proportions ± 95% confidence intervals. To test whether higher prey mortality occurred even in absence of apparent signs of predation, the status of the prey (dead or alive) was compared between treated replicates and their matched controls using the McNemar mid-p test [48] in the R software [49]. The occurrence of predation among treatments (type of prey) after 12 and 24h was compared using Fisher’s exact test followed by pairwise comparisons with Benjamini-Hochberg adjustment to control the false discovery rate (FDR). True difference between predation choices was investigated using a binomial two-sample test of proportions in R. Data of the *in vivo* predation test were analyzed using the proc mixed procedure in SAS University Edition [50]. The normality of residuals was achieved, so a repeated measures analysis of variance (ANOVA) with autoregressive correlation structure was performed to compare differences of brood survival (number of eggs and surviving larvae and pupae) due to treatment, brood stage (post-oviposition time) and their interaction. Results are presented as percentages of brood survival (number of surviving larvae or pupae x 100 / initial number of eggs). Regarding *S*. *scimitus* predation assessment of phoretic *Varroa* mites, a log-rank Kaplan-Meier survival analysis was carried out to compare the survival curves of the *Varroa* in the presence or the absence of the predatory mite (survival package in R). *Varroa* death events that occurred on the same day as their respective bee death were considered as right censored data. Significance was defined as p ≤ 0.05 for all analyses.

## Results

### *In vitro* assessment of *S*. *scimitus* predation upon *V*. *destructor* and bee brood

**Fig 1:**
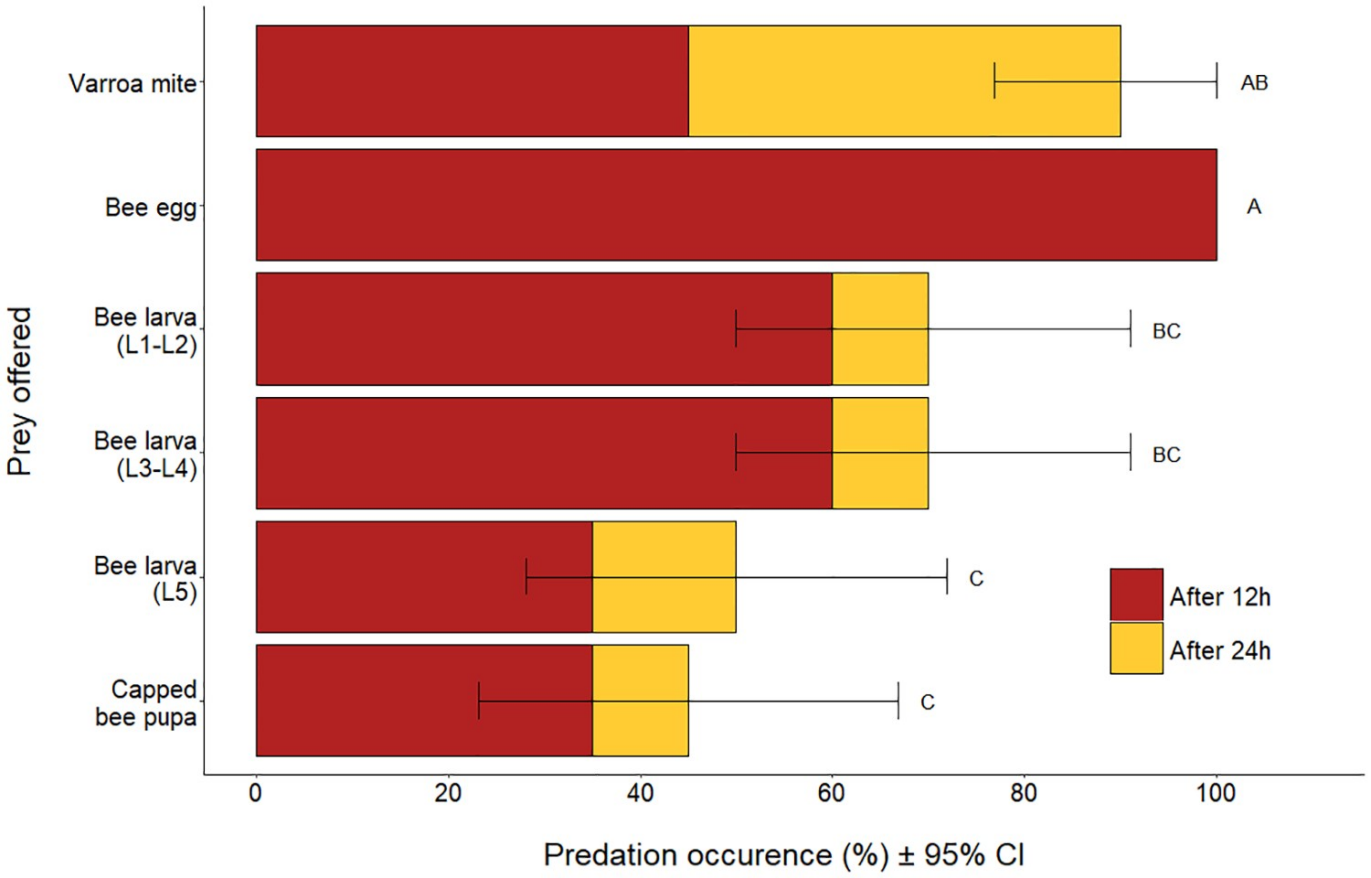
Occurrence of predation of *Varroa destructor* (female adults) and five different honey bee brood stages by the predatory mite *Stratiolaelaps scimitus*, after 12h and 24h of confinement in experimental arenas. Each arena (n = 20 per type of prey) contained 20 starved female predatory mites and a single prey. Error bars show the 95% confidence intervals after 24 h. Different letters represent significant differences (p ≤ 0.05, Fisher’s exact test followed by pairwise comparisons with Benjamini-Hochberg adjustment) in predation occurrence at the end of the test.

**Fig 2:**
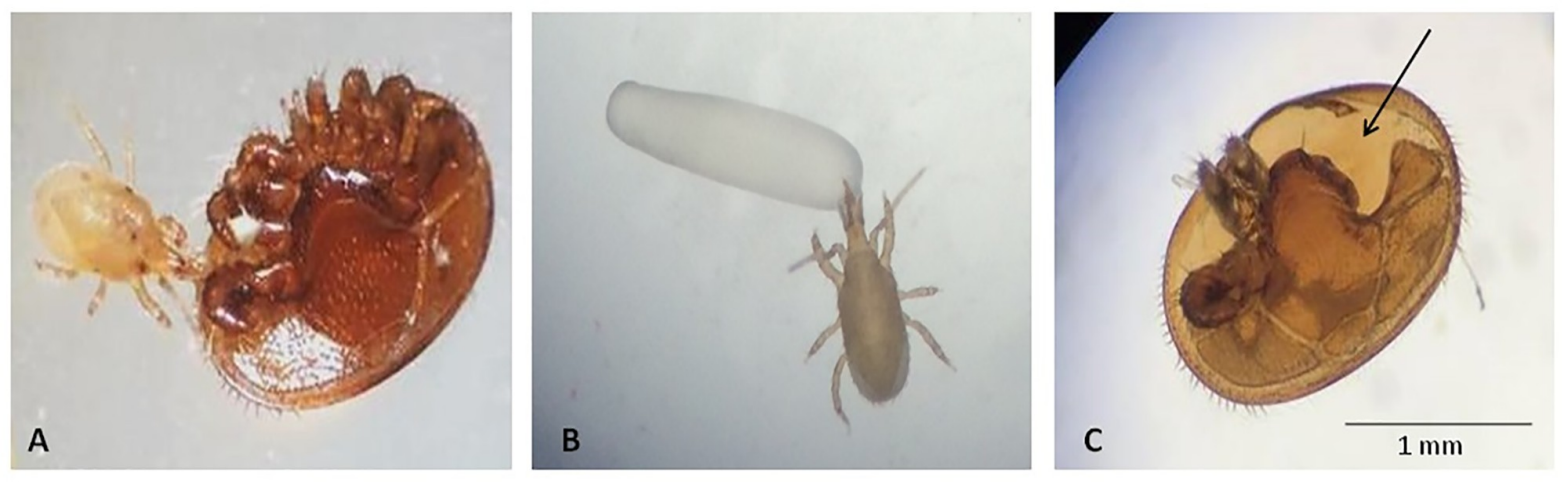
**The predatory mite *Stratiolaelaps scimitus* feeding on a female *Varroa* mite (A) and a honey bee egg (B) under laboratory conditions. After being attacked by *S*. *scimitus*, the *Varroa* showed characteristic signs of predation (C) such as missing legs and holes in the cuticle (arrow)**. (Photos: Sabrina Rondeau, 2016).

Predation occurred on all types of prey offered to *S*. *scimitus* (Fig 1). Only the prey with obvious signs of predation were recorded as having been predated upon (Table 1). This includes live observations of predation or attack, eggs fully consumed, liquefied larvae and *Varroa* mites with obvious missing appendages and damaged cuticle. Obvious predation events (stylet inserted into the body of the prey) were observed in real time at least twice for each type of prey (Fig 2). At the end of the experiment, an average of 15 ± 3 (mean ± SD) predatory mites were still alive in each arena.

**Table 1:** Status of *Varroa destructor* (female adults) and five different honey bee brood stages after a maximum of 24h of confinement with *Stratiolaelaps scimitus* under laboratory conditions. Each arena (n = 20 per type of prey) contained 20 starved female predatory mites and a single prey.

Prey /state	Number of observations (n)				
	Fully consumed	Alive with predation	Alive without predation	Dead with predation	Dead without predation
*Varroa* mite	0	2	0	16	2
Bee egg	20	0	0	0	0
Bee larva (L1-L2)	0	0	1	14	5
Bee larva (L3-L4)	0	1	4	13	2
Bee larva (L5)	0	6	10	4	0
Capped bee pupa	0	1	4	8	7

All *Varroa* mites in the control group were still alive at each observation period. Similarly, all honey bee eggs in the control group were still present and intact after 12h, while the eggs of the group treated with *S*. *scimitus* were all fully consumed at that same time. Analysis of the status of honey bee larvae and pupae between treated replicates and their matched controls revealed that mortality of honey bee brood likely occurred more often in presence of *S*. *scimitus*, regardless of the presence (mid-p < 0.001, McNemar test) or the absence (mid-p = 0.013, McNemar test) of apparent signs of predation (S1 Table). In this analysis, data of all instars of bee larvae and pupae have been pooled together to obtain a larger sample size for statistical purposes.

During the first 12h of confinement with *S*. *scimitus*, obvious predation events occurred significantly more often for honey bee eggs than for the other groups of prey (Fisher’s exact test, p < 0.001; FDR adjusted p < 0.010). At the end of the test, the overall occurrence of predation differed significantly between the type of prey offered to *S*. *scimitus* (Fisher’s exact test, p < 0.001; Fig 1), with the bee eggs and the *Varroa* mites being predated more frequently. The 5^th^ bee larval instar and the capped bee pupae showed the lowest occurrences of predation, which were significantly less than those of bee eggs (FDR adjusted p’s ≤ 0.002) and *Varroa* mites (FDR adjusted p’s ≤ 0.050) although not significantly different from L1-L2 and L3-L4 larvae (FDR adjusted p’s ≥ 0.353). The occurrence of predation in L1-L2 and L3-L4 larvae differed significantly only from that of bee eggs (p’s = 0.050).

### Prey preference test

**Fig 3:**
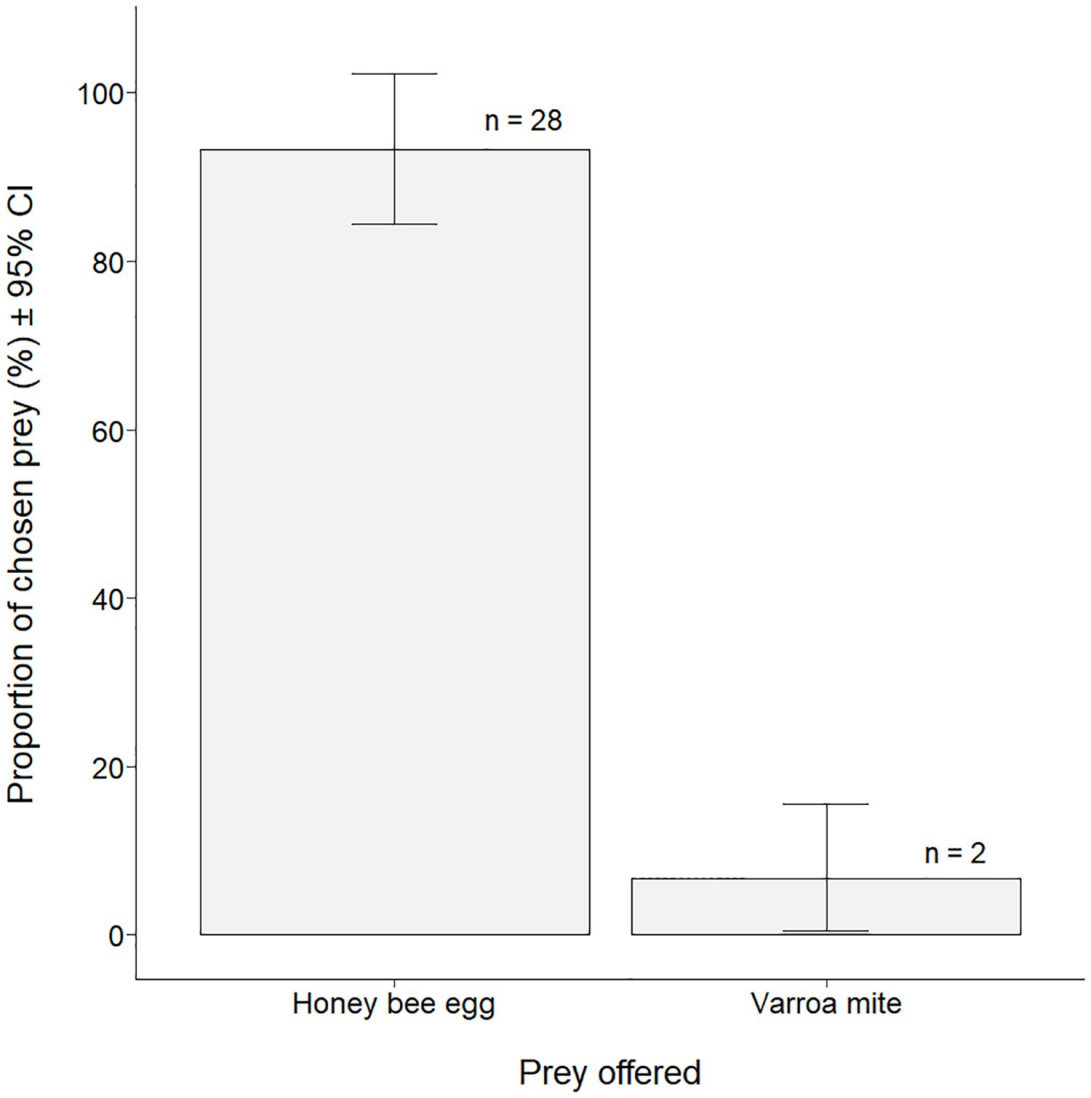
Proportion of honey bee eggs and *Varroa* mites first chosen by *Stratiolaelaps scimitus* during a preference test where both prey were offered simultaneously (n = 30) to ten starved *S*. *scimitus* individuals.

When both prey were offered simultaneously, *S*. *scimitus* individuals first predated upon the bee egg (n = 28) over the *Varroa* mite (n = 2) significantly more often (Fig 3; binomial test, n = 30, p < 0.001). In most cases (25/28), the bee egg was consumed during the first hour while the predation upon the *Varroa* only occurred after 4 or 5 hours. In this last scenario, the bee egg remained untouched while the *Varroa* was dead and showed evident signs of predation (multiple missing appendages). Predation of both prey never occurred during the same one-hour observation interval.

### *In vivo* assessment of *S*. *scimitus* predation upon bee brood

**Fig 4:**
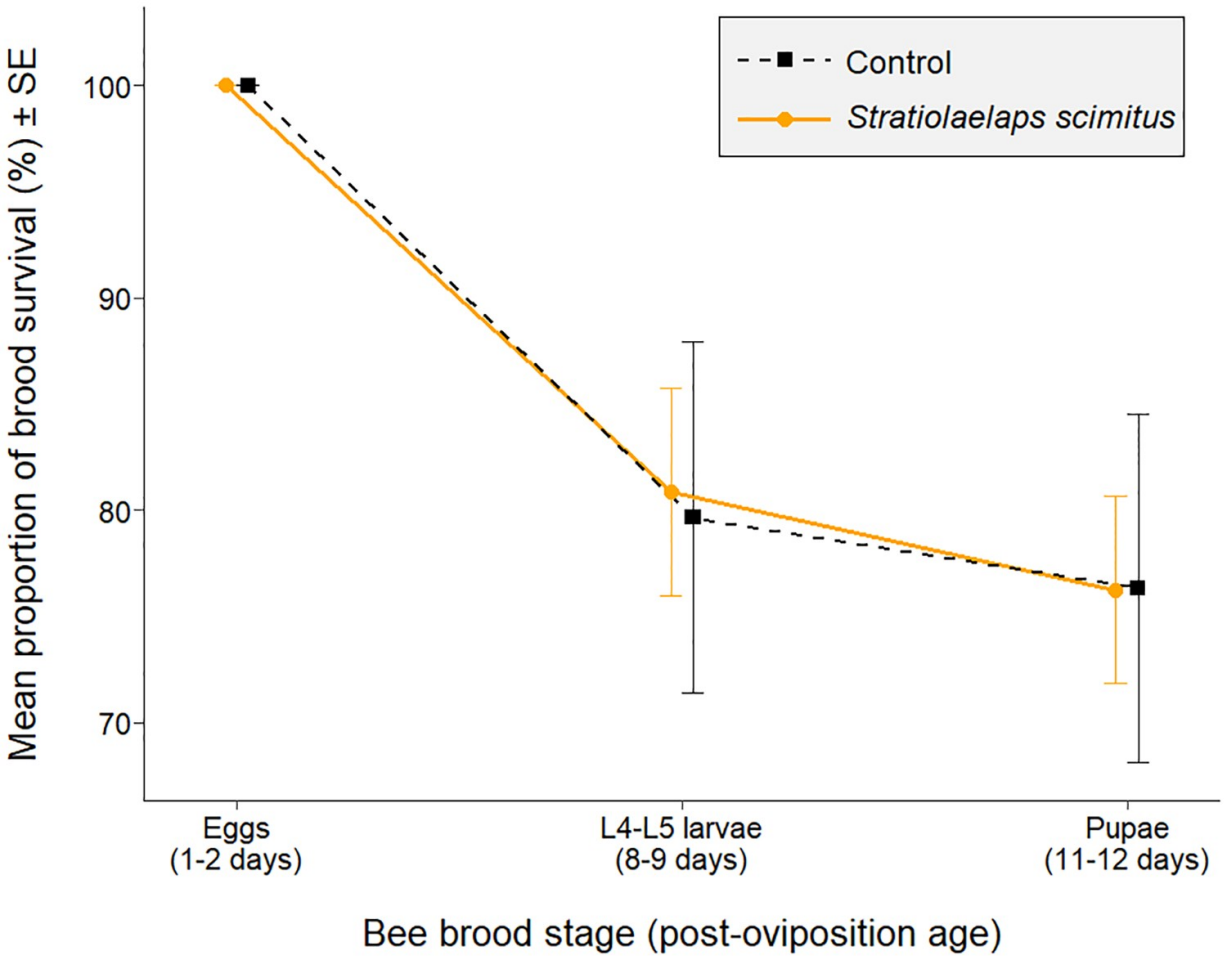
Effect of the inoculation of honey bee colonies (n = 5) with ≈ 12,500 *Stratiolaelaps scimitus* individuals on the mean proportion of bee brood survival from the eggs to the pupae in comparison with untreated colonies (control; n = 3). On average, 1800 ± 111 (mean ± SE) eggs have been marked in each colony and monitored over time (August 09 to 21, 2017). There was no effect of the treatment on the bee brood survival (repeated measures ANOVA; F(1,6) = 0.03, p = 0.864).

Two colonies in the control group were rejected from the analysis due to abnormally low brood survival (0 and 23%) between the first two periods of data collection (i.e., before reaching the L4-L5 larval stage). On average, 1800 ± 111 (mean ± SE) eggs were marked in each colony and monitored over time. The initial number of eggs did not differ between groups (two sample t(6) = 0.103, p = 0.922). The repeated measures ANOVA revealed no interaction between treatment and time (F_(2,12)_ = 0.05, p = 0.956) and there was no significant effect of the treatment (F_(1,6)_ = 0.03, p = 0.864) on the bee brood survival. Only the time had an effect on the brood survival (F_(2,12)_ = 21.92, p < 0.001) with an average survival (mean ± SE) of 79.7 ± 8.3% and 80.9 ± 4.9% of the L4-L5 larvae and 76.3 ± 8.2% and 76.3 ± 4.4% of the pupae for the control and the treated colonies respectively (Fig 4).

### *S*. *scimitus* predation of phoretic *Varroa* mites

**Fig 5:**
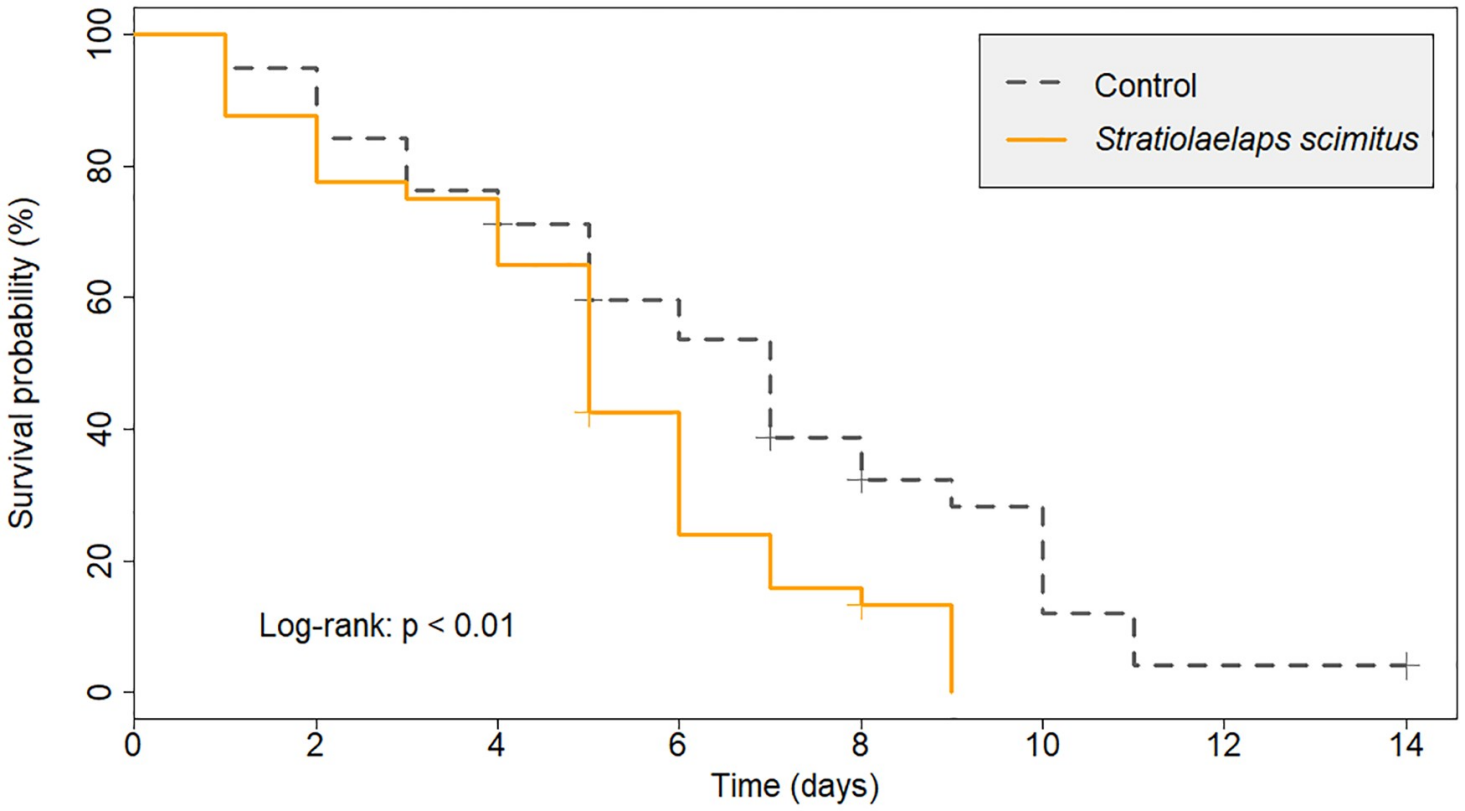
Kaplan-Meier survival curves of the phoretic *Varroa* mites when confined in experimental arenas with 20 starved *Stratiolaelaps scimitus* individuals (n = 40) or none (control; n = 38). Each arena consisted of a modified plastic pill bottle and contained one worker bee parasitized by a single *Varroa* mite. Death events of both the *Varroa* and the bee have been recorded once a day and *Varroa* death events that occurred on the same day as their respective bee death were considered as right censored data.

Some *S*. *scimitus* individuals escaped and were found in two control arenas which were rejected. The log-rank Kaplan-Meier survival test showed a significantly lower survival rate of *Varroa* mites when *S*. *scimitus* was present (Fig 5; p < 0.01). Mortality of 90% of phoretic *Varroa* in control and treated arenas occurred after ten days and eight days respectively. No *Varroa* mite survived longer than nine days in the presence of the biocontrol agent. On the other hand, we stopped monitoring the survival of the last *Varroa* mite in the control group after 14 days and artificially killed it by freezing (right censoring). Within the treated group, all *Varroa* mites that were found dead showed signs of predation (missing legs or mouthparts, holes in the cuticle, etc.). An average of 9 ± 4 (mean ± SD) predatory mites were still alive in each treated arena at the end of the test.

## Discussion

Our experiment indicates that, under controlled conditions, *S*. *scimitus* attacks and feeds upon *Varroa* mites when no other food choice is given. Despite the relatively smaller size of *S*. *scimitus* compared to the *Varroa* (Fig 2), the predator still succeeded in killing them. This is not surprising considering that *S*. *scimitus*, like the other mites of the family Lealapidae, is an aggressive edaphic predator [51]. Typically, *Varroa* mites that had been attacked by *S*. *scimitus* showed many missing legs and large holes in their cuticle. This is typical of the attack of many mesostigmatan mites that strive at the leg joint of large arthropods until the hemolymph flows [51]. In the experimental arenas, the predators were constantly on the move, searching for prey. However, as they are used to live in the soil, they were mainly active and searching in the vermiculite at the bottom of the vial, climbing the walls only from time to time. On the opposite, most of the time the *Varroa* remained hidden on the piece of Nitex^®^ cloth that served as a cover. This could explain why half of the predation events occurred only after 12 hours despite the small size of the arena and the relatively high number of starved predators it contains. We observed some group attack events, but attacks by a single mite were also common. During a group attack, the *Varroa* mite was first found and targeted by a single *S*. *scimitus* individual before being rapidly surrounded by others and assailed with quick jabs with the chelicerae. Then, the *Varroa* was presumably drained of its fluids (considering the feeding behaviour of *S*. *scimitus*) and the cuticle, apparently empty, was left behind.

Under these same restrictive laboratory conditions, *S*. *scimitus* was able to feed upon every honey bee developmental stages from egg to pupa. This goes against our predictions, which were based on the facts that predation of sciarid eggs and pupae by *S*. *scimitus* rarely occurs as the predatory mite is thought to prefer mobile stages and smaller prey [34, 52]. In fact, the 4^th^ instar larvae of the sciarid flies are not always attacked by *S*. *scimitus* because they are presumably too large (up to 7 times the size of the adult mite), as postulated by Wright and Chambers [34]. These are similar in size and weight to the honey bee 2^nd^ or 3^rd^ larval instar [47, 53], which in the present case were repeatedly attacked. All bee eggs were completely consumed by *S*. *scimitus* while the larvae were almost exclusively attacked at their body ends (head or anus). Some pupae were also attacked despite being protected by a sealed wax cell. However, we do not know whether these cells had been previously damaged during their sampling, allowing the mites to enter the cell through small openings, or if the predators punctured the wax by themselves. Group feeding was the norm for all types of prey. Usually, the prey was initially attacked by a single mite before others joined it and began to feed. Here, chemical cues could be involved [34].

In hindsight, our results are not so surprising if we consider the specific and highly restrictive conditions of our test. In fact, a single prey was given to multiple highly polyphagous predators that had been starved for 48 hours, without alternative food sources. These conditions were put in place specifically to ensure that any potential predation by the predator would be detected, even though these are unrealistic of in-hive conditions. The biggest difference between the conditions of both environments was the accessibility of prey. Within the bee colony, eggs and larvae are found in cells and are cared for and protected by worker bees. On the opposite, in our experiment, the brood was unprotected and offered to the predatory mites in a restricted environment so that their presence was easily detectable. Thus, these results should be taken with caution as predation tests conducted in the colony prove to be more realistic and revealing of the predation behavior of *S*. *scimitus* and the non-target effects that might ensue.

When a choice is given under controlled conditions, *S*. *scimitus* first predates upon the unprotected honey bee egg over the free *Varroa* mite. Since these two prey were the most consumed in the previous trial, it was relevant to assess the predator’s preference when both prey are present, as this is the case in a bee colony. In many cases, even if a prey has been contacted by a predator, the decision to attack may be influenced by the assessment of relative risks and costs compared with the nutritional benefits brought by the prey at hand [54]. Here, the smaller size of the bee egg and its soft body certainly make it easier for *S*. *scimitus* to attack compared with the *Varroa*. The *Varroa* ability to flee the predator also plays a role. Indeed, this escape behavior might explain why the time elapsed before the predation event was much longer when the *Varroa* was predated first than when the bee egg was.

Interestingly, when introduced inside colonies, *S*. *scimitus* does not have negative effects on the survival of the honey bee brood. This suggests that the predatory mite does not feed upon the bee brood inside the colony. Here, there are two possible explanations. First, the tendency of *S*. *scimitus* to seek and stay in the vermiculite or other debris and the protection provided by the worker bees may be sufficient to prevent the predator from attacking the brood. Indeed, the ecology of *S*. *scimitus* (i.e., soil-dwelling predator) leads us to believe that the predator rather tends to search for prey at the bottom of the hive, where the debris are, than at the center of the bee cluster where the brood is. Observations made in the colonies three days after the introduction of the predator seem to confirm this behavior since several predators were found at the bottom of the hive while very few were observed walking on the brood frames. The displacement of vermiculite by the bees in the hive certainly contributed to the mites’ dispersal since much of the vermiculite was moved to the bottom of the hive over time. Presumably, this propensity to seek debris may also limit the predator’s ability to attack *Varroa* mites within colonies, as the adult parasites are mainly phoretic or in the brood cells. We know that *S*. *scimitus* remained in the colony for at least ten days, since we observed its presence in the debris at the bottom of the hive and confirmed it under magnification. Moreover, the invasion of a brood cell by *S*. *scimitus* is likely to result in the removal of the mite by worker bees during routine maintenance duties, preventing the brood from being predated [28]. A second explanation for the absence of bee brood predation in the colony would be the presence of other food sources. During our observations, we collected debris in the bottoms of hives for screening purposes. In addition to *Varroa* mites, we recorded the presence of various species of mites and spiders, springtails, ants, nitidulid beetles and wax moth larvae. There were also plenty of mold mites (presumably *Tyrophagus putrescentiae*) which were most likely introduced with the biocontrol agent since they are supplied as food with the predatory mite during the transit and in storage. Thereby, the presence of multiple alternative food sources might prevent non-target effects on the bee brood, while also reducing the efficiency of *S*. *scimitus* to target the *Varroa*.

Assessing the risk of honey bee brood predation by *S*. *scimitus* is a step that should be taken seriously, considering the deleterious impacts that this predation could have on the strength and the survival of the colony. Based on previous observations conducted in Canada, biocontrol suppliers currently suggest using 150 to 200 ml of the *S*. *scimitus* mixture for *Varroa* control [42, 43]. In our *in-vivo* trial, we used 500 ml of this mixture (12,500 individuals) and considering the voracity of the predator, we believe this must be enough to detect a predation effect if there is any. We acknowledge that the number of replicates used in this trial would have benefited from being higher. Nevertheless, our results correspond to those obtained using observation hives, which reinforce the reliability of our findings (S1 Appendix). In these undescribed tests, we introduced hundreds of starved *S*. *scimitus* individuals in observation hives containing a single frame of brood and we observed their behavior for several hours, using a red light in the dark. When worker bees were absent, most of the mites remained in the vermiculite poured on top of the frame but some of them occasionally walked on the comb. Some mites were observed entering brood cells containing a bee egg, but predation was rarely observed. Moreover, when worker bees were present in the observation hives, the mites did not climb on the frames at all and no brood predation was observed. In addition to corroborating the absence of significant predation risk of the bee brood by *S*. *scimitus* within colonies, these observations also support the role of worker bees in brood protection.

Observations made in laboratory revealed that *S*. *scimitus* individuals do not attack *Varroa* mites when they are attached to the body of bees. Indeed, even when the predatory mites were deposited carefully with a small paint brush on the body of an adult worker bee, these did not adhere to the insect body and fell to the slightest bee movement. Moreover, *S*. *scimitus* has never been recorded to be phoretic, as most of the lealapid mites [55]. Even if the predatory mite is known to be able to feed upon phoretic hematophagous mites in infested birds and lizards [41, 46], it seems that it only attacks the parasites when they are off their host body [40].

Since the biocontrol agent under study is not able to attack phoretic *Varroa* mites, it is unlikely that it will be effective enough to be used alone in *Varroa* control. When ready to reproduce, the female *Varroa* mite leaves its honey bee host to invade a worker cell approximately 20h before its capping [56] and the entire reproductive cycle takes place into that cell. Thus, the effective period for *S*. *scimitus* to enter into the brood cell in tandem with the *Varroa* is short, which makes it unlikely that the predador will impact significantly neither on reproductive adult *Varroa* mites nor on *Varroa* eggs or larvae [28]. After this period of time, reproductive *Varroa* mites are blocked by the brood cell cap and only the phoretic parasites remain accessible to *S*. *scimitus*. Thereby, to be at least partially effective, the biocontrol agent must be able to search bee bodies for adult *Varroa* mites and attack them. Likewise, most of the chemicals used in *Varroa* control only kill the phoretic mites, except for formic acid which effectively kills *Varroa* mites in sealed brood cells [57].

In our trial, however, all *Varroa* mites that had fallen from their bee host body were predated upon by *S*. *scimitus* and died in less than 24h. It strongly suggests that *S*. *scimitus* only predates upon *Varroa* mites that naturally fell from the bees. In fact, a certain percentage of mites in a colony simply lose their grip and fall to the bottom of the hive over time. Moreover, in order to avoid parasitism by *V*. *destructor*, honey bees often exhibit defensive behaviors such as “grooming” which involves self-removal of phoretic *Varroa* mites on the body of adult bees [58]. When effective, this behavior leads to the removal of the parasite which is more likely to fall on the hive floor. In our experiment, the reduced probability of survival recorded for the phoretic *Varroa* mites from the treated group is due to the fact that the *Varroa* were instantly attacked by *S*. *scimitus* after a natural fall from their host body. In the control group, fallen *Varroa* mites survived longer and even had a chance to return on their host body.

As previously mentioned, *S*. *scimitus* is very unlikely to provide effective *Varroa* control if used alone. However, in future assessments, it might be interesting to test its potential when combined with other existing methods or new avenues in a context of integrated pest management. We demonstrated that instead of attacking phoretic *Varroa* mites, *S*. *scimitus* is more likely to predate upon the mites that fall on the bottom of the hive. In doing so, the biocontrol agent might have a similar effect to that of screen bottom boards or might increase their effectiveness in a similar way than sticky sheets [59]. We know that about 50% of the *Varroa* are still alive and very active when they fall on the hive floor [59]. Thereby, screen bottoms boards that allow *Varroa* to fall through it are often used to prevent the living fallen mites from returning to the colony. Even if not reliable as a single control technique, the use of these screen boards could reduce about 20% of the mite population over the season and increase the degree of *Varroa* control obtained with soft chemicals and other cultural practices [27, 60, 61]. In parallel, Reinbacher et al. [62] recently showed that the entomo-pathogenic fungus *Metarhizium anisopliae*, in addition to its lethal effect on *Varroa* mites, is repelling the parasite from attaching to bees. Interestingly, the fungus is known to be harmless to *S*. *scimitus* and the combination of *M*. *anisopliae* and *S*. *scimitus* have been shown to improve the efficacy of the predator against pupating western flower thrips in container studies [63]. Therefore, assessing the combined effect of both agent in *Varroa* control might be an avenue of interest. Whether the introduction of *S*. *scimitus*, alone or in combination with *M*. *anisopliae*, would be more effective, convenient or cheaper than the combined use of bottom boards and sticky sheets is, however, uncertain and is worth more consideration.

In a recent study [42], Rangel and Ward showed, using *in vitro* assays, the capacity of *S*. *scimitus* in attacking free *Varroa* mites but they raised questions regarding the overall ability of the predator and whether it could prey on honey bee brood. Here, not only did we bring answers to several of their questions, but we provided additional, crucial information on *S*. *scimitus* as a biocontrol agent of *Varroa* mites. For instance, by using more realistic conditions under which we conducted our *in vitro* predation tests (32°C; 70% RH vs 29.5°C; uncontrolled humidity in [42]), we showed that *S*. *scimitus* can survive and be active within the range of temperature and humidity conditions of a honey bee colony [64]. Since free *Varroa* mites are uncommon in the hive, our study also provides a better understanding of the limitations of *S*. *scimitus* in controlling *Varroa* mites under more realistic conditions.

In summary, our study provides evidence that *S*. *scimitus* does not represent a significant threat to the honey bee brood but suggests that its effect in *Varroa* control will probably be limited as it does not attack phoretic *Varroa* mites. Our results represent an important step in assessing the potential of *S*. *scimitus* to control *V*. destructor and provide novel information about the behavior of the predator inside the honey bee colony. Nevertheless, the actual efficacy of the predatory mite to control *Varroa* populations in honey bee colonies still needs to be investigated in greater depth. As *S*. *scimitus* is highly polyphagous, assessing the predator’s ability to control other honey bee pests found on the hive floor, such as wax moth and small hive beetle larvae, should also be considered.

## Supporting information

S1 Fig Inoculation of a honey bee hive with *Stratiolaelaps scimitus*. (JPG)

S1 Table Paired comparison of the status of honey bee brood (larvae and pupae) between treated and control groups. (DOCX)

S1 File Information file for S1, S2, S3 and S4 Datasets. (DOCX)

S1 Dataset Data from our *in vitro* predation test. (CSV)

S2 Dataset Data on *S*. *scimitus* prey preference. (CSV)

S3 Dataset Data from our *in vivo* predation test. (CSV)

S4 Dataset Data on *S*. *scimitus* predation of phoretic *Varroa* mites. (CSV)

S1 Appendix Additional monitoring using observation hives. (DOCX)
