# Supplementary figures and images for: Risk assessment and predation potential of Stratiolaelaps scimitus (Acari: Laelapidae) to control Varroa destructor (Acari: Varroidae) in honey bees
Source: PLoS One. 2018 Dec 7;13(12):e0208812. doi: 10.1371/journal.pone.0208812 (PMC6286145; doi:10.1371/journal.pone.0208812)

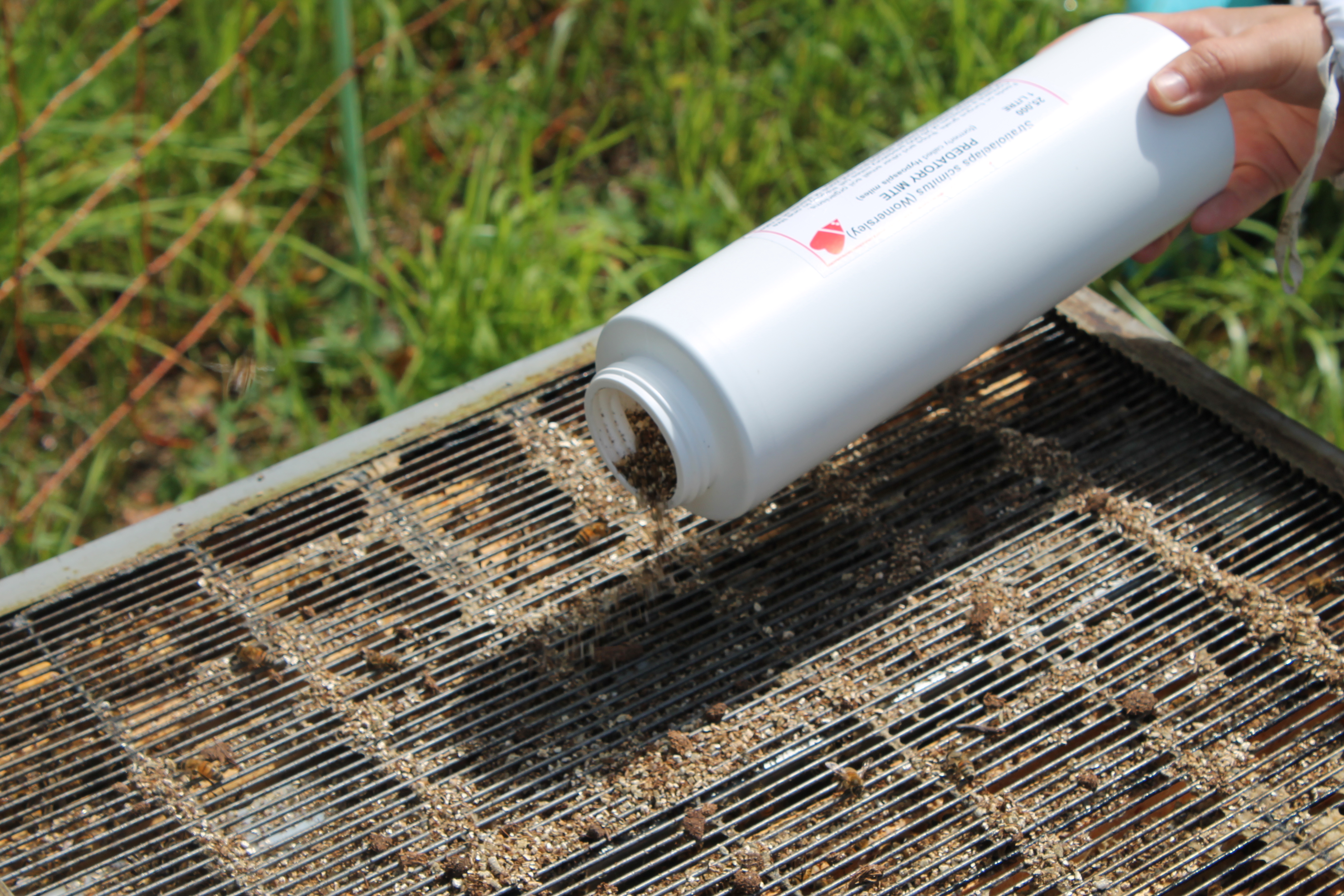

Supplement: S1 Fig — (JPG) [file pone.0208812.s001.JPG]
