# Supplementary material for: Risk assessment and predation potential of Stratiolaelaps scimitus (Acari: Laelapidae) to control Varroa destructor (Acari: Varroidae) in honey bees
Source: PLoS One. 2018 Dec 7;13(12):e0208812. doi: 10.1371/journal.pone.0208812 (PMC6286145; doi:10.1371/journal.pone.0208812)
Supplement: S1 Table — (DOCX) [file pone.0208812.s002.docx]

**S1 Table. Paired comparison of the status of honey bee brood (larvae and pupae) between treated and control groups.** The number of observations is based on the presence or the absence of apparent signs of predation of the brood in the treated group after 12 or 24h of confinement with *Stratiolaelaps scimitus* in experimental arenas.

| **Status of matched pairs* of honey bee larvae or pupae** | | **Number of observations (n)** | | | | | |
| --- | --- | --- | --- | --- | --- | --- | --- |
|  |  | **No signs of predation** | | | **Apparent signs of predation** | | |
| **Treated** | **Control** | **12h** | **24h** | **Total^a^** | **12h** | **24h** | **Total^b^** |
| Alive | Alive | 22 | 18 | 40 | 8 | 0 | 8 |
| Alive | Dead | 3 | 0 | 3 | 0 | 0 | 0 |
| Dead | Alive | 9 | 4 | 13 | 22 | 8 | 30 |
| Dead | Dead | 8 | 10 | 18 | 8 | 1 | 9 |

### * A pair refers to one honey bee larva or pupa of the treated group and its respective control counterpart; Treated = bee brood confined with the predatory mite *S. scimitus*; Control = bee brood unexposed to the predatory mite.

### ^a^ McNemar test: mid-p value = 0.013

^b^ McNemar test: mid-p value < 0.001
