# Supplementary material for: Risk assessment and predation potential of Stratiolaelaps scimitus (Acari: Laelapidae) to control Varroa destructor (Acari: Varroidae) in honey bees
Source: PLoS One. 2018 Dec 7;13(12):e0208812. doi: 10.1371/journal.pone.0208812 (PMC6286145; doi:10.1371/journal.pone.0208812)
Supplement: S1 File — (DOCX) [file pone.0208812.s003.docx]

Information file for datasets of the manuscript entitled “Risk assessment and predation potential of *Stratiolaelaps scimitus* (Acari: Laelapidae) to control *Varroa destructor* (Acari: Varroidae) in honey bees”.

The data were collected by S. Rondeau. Please contact Prof. V. Fournier if you have any questions (valerie.fournier@fsaa.ulaval.ca).

**S1 Dataset. Data from our *in vitro* predation test**

The file contains the following data columns:

- Date (Date at which the test was conducted, July 21 to September 1, 2016)
- Time (Time at which the sample was placed in the incubator)
- Prey (varroa = adult female varroa mite; egg = honey bee egg; L1-L2 = honey bee 1^st^ or 2^nd^ bee larval instar; L3-L4 = 3^rd^ or 4^th^ bee larval instar; L5 = 5^th^ bee larval instar; pupa = capped bee pupa0
- Tx (Treatment: control = arena with a prey but without predators; Sscimitus = arena with a prey and 20 starved adult female *S. scimitus*)
- Prey_tx (Combination of variables “Prey” and “Tx”: **control** (varroa_ctr = adult female varroa mite; egg_ctr = honey bee egg; L1-L2_ctr = honey bee 1^st^ or 2^nd^ bee larval instar; L3-L4_ctr = 3^rd^ or 4^th^ bee larval instar; L5_ctr = 5^th^ bee larval instar; pupa = capped bee pupa) OR **Sscimitus** (varroa = adult female varroa mite; egg = honey bee egg; L1-L2 = honey bee 1^st^ or 2^nd^ bee larval instar; L3-L4 = 3^rd^ or 4^th^ bee larval instar; L5 = 5^th^ bee larval instar; pupa = capped bee pupa)
- Replicate (1 to 20 for treatments with predator; 1 to 15 for controls)
- ID (sample identification)
- Temp. (Temperature of the incubator when the sample was placed in it, in °C)
- RH (Relative humidity, in %)
- Status_12h (Status of the prey after 12h: fully consumed, dead or alive, with or without predation)
- Status_24h (Status of the prey after 24h; NA = predation already occurred after 12h)
- Predation_overall (yes = predation occurred within 24h; no = predation did not occur)
- Living_Ss (Number of living *S. scimitus* individual recorded at the end of the trial)
- Dead_Ss (Number of dead *S. scimitus* individual recorded at the end of the trial)
- Total_Ss (Total number of *S. scimitus* recorded at the end of the trial)

**S2 Dataset. Data on *S. scimitus* prey preference**

The file contains the following data columns:

- Date (Date at which the test was conducted: August 5, 12 and 19, 2016)
- Time (Time at which the samples were placed in the incubator)
- Replicate (1 to 30)
- Temp. (Temperature of the incubator when the sample was placed in it, in °C)
- RH (Relative humidity, in %)
- Order (Randomized order of prey introduction in the arena; varroa or bee egg first)
- Choice (First prey predated by *S. scimitus*: varroa mite or honey bee egg)
- Duration (Time elapsed before the predation event; in h)
- Living_Ss (Number of living *S. scimitus* individual recorded at the end of the trial)
- Dead_Ss (Number of dead *S. scimitus* individual recorded at the end of the trial)
- Total_Ss (Total number of *S. scimitus* recorded at the end of the trial)

**S3 Dataset. Data from our *in vivo* predation test**

The file contains the following data columns:

- Date (Date at which the test was conducted, August 11 to 21, 2017)
- Treatment (control = untreated colonies; Sscimitus = colonies treated with the predatory mite *Stratiolaelaps scimitus*)
- ID (hive identification)
- Time (Monitoring period: T0 = egg monitoring ; T1 = larvae monitoring; T2 = pupae monitoring)
- Brood_A (Number of eggs, larvae or pupae in cells of the first side of the brood frame)
- Brood_B (Number of eggs, larvae or pupae in cells of the second side of the brood frame)
- Brood_total (Total number of eggs, larvae or pupae in cells of the brood frame)

**S4 Dataset. Data on *S. scimitus* predation of phoretic varroa mites**

The file contains the following data columns:

- Date (Start date of the test, July 10 or August 9, 2017)
- Treatment (control = untreated arenas; Sscimitus = arenas treated with 20 female *Stratiolaelaps scimitus*)
- ID (arena identification)
- Censoring (whether the data is right censored: 0 = censored data ; 1 = uncensored data)
- Survival (Survival time of the varroa, in days)
- Predation (Whether predation of the varroa had occurred (yes) or not (no))
- Living_Ss (Number of living *S. scimitus* individual recorded at the end of the trial)
- Dead_Ss (Number of dead *S. scimitus* individual recorded at the end of the trial)
- Comment (Includes relevant information about censoring or contamination)
