# Supplementary material for: Risk assessment and predation potential of Stratiolaelaps scimitus (Acari: Laelapidae) to control Varroa destructor (Acari: Varroidae) in honey bees
Source: PLoS One. 2018 Dec 7;13(12):e0208812. doi: 10.1371/journal.pone.0208812 (PMC6286145; doi:10.1371/journal.pone.0208812)
Supplement: S1 Appendix — (DOCX) [file pone.0208812.s008.docx]

Additional monitoring of *S. scimitus* predation upon bee brood

using observation hives

Methods

In addition to the trial described for the “*in vivo* assessment of *S. scimitus* predation upon bee brood”, further observations were made using an observation hive. Those observations were made at three different periods of time in May and June 2017.

The same home-made wooden observation hive was used throughout the tests. Glass frames at both sides of the hive allowed observations of a single Langstroth-style deep frame. Two screened moisture vents were present, one at each narrow side of the hive.

Brood frames and honey bees were obtained from the livestock of the Centre de recherche en sciences animales de Deschambault (CRSAD).

The predatory mites *Stratiolaelaps scimitus* were starved individually for 48h in small portion containers (1 oz) with a piece of moistened tissue paper prior to their transfer in the hive.

**May 2, 2017**

One frame of brood containing eggs, larvae and capped pupae and covered with worker bees was inserted in the observation hive. A thick layer of petroleum jelly (Vaseline®) was spread around air vents to prevent mite escape. The hive was placed in a dark, warm and humid room (28°C, 40% RH; complete darkness) throughout the test.

About 600 starved predators were then transferred to 150 ml of pre-autoclaved moistened vermiculite and poured on top of the frame. Using headlamps with red light, observations were made in the dark for two hours following the introduction of the mites, and for four hours/day during the next three days (two hours each in the morning and in the afternoon). Observations were made by two observers, one for each side of the frame, for a total observation period of 28 hours. The behavior of both the mites and the bees was monitored, including the movements of *S.*

*scimitus* in the hive and on the frames, the attempts of brood predation by *S. scimitus* and the behavior of bees toward predators.

**May 30, 2017**

One frame of brood containing mostly eggs was inserted in the observation hive without bees. Prior to observations, a total of 60 cells containing an egg were marked using a permanent marker on a transparent sheet of acetate placed on each side of the frame. Only freshly laid eggs (standing up in the cell) were marked. About 1,000 starved mites were transferred to 75 ml of moistened vermiculite and poured on top of the frame. We used the same observation protocol as previously described. After two days, we checked with previous acetates if the eggs (or freshly hatched larvae) were still present. Cells with a missing egg were marked with a permanent marker of another color and the frame was returned to the hive. This time, the hive was kept in a growth chamber (32°C, 75°C, and complete darkness) between observation periods. Observations were made in the same room as previously described.

**June 14, 2017**

One frame of brood containing mostly eggs was inserted in the observation hive without bees. About 1,500 starved mites were transferred to 75 ml of moistened vermiculite and poured on top of the frame. We used the same observation protocol as previously described, for the same period of time and in the same physical conditions.

**Results**

**May 2, 2017**

Fifteen minutes following the introduction of the mites, almost all the vermiculite had fallen to the bottom of the hive due to bees’ attempt to remove it. The predatory mites were active but stayed in the vermiculite at the bottom of the hive throughout our observations. Most of the time,

when *S. scimitus* individuals undertook to climb on the frames or the walls of the hive, they fell back to the bottom due to the movements of bees. The same behavior was recorded during the next three days. Bees, for their part, acted normally. They continued to take care of the brood (feed the larvae, cap the cells, ventilate) and did not seem bothered by the presence of the mites. Only 2 bees died over the observation period.

**May 30, 2017**

Mites stayed in the vermiculite on top of the frame, walking on the comb only occasionally and for a short period of time (< 10 minutes). We observed some mites getting into brood cells containing an egg, but the eggs were usually not attacked – at the opposite of our tests in lab. During our 28h observation period, we observed only one predation event of a bee egg by *S. scimitus*. Some mites were recorded drowned in honey. After two days, only five of the sixty marked eggs were missing (8.3%). We don’t know if those eggs had been predated or if they dried out.

**June 14, 2017**

Even if more mites were added to the hive, the predator behavior did not change. The mites remained mostly in the vermiculite and no predation was observed. There appears to have been some mite escape or mortality since few predators (dead or alive) were recorded during the last observation day.
